# The Current Landscape of Pancreatic Cancer Management in Sub‐Saharan Africa – A Perspective Review

**DOI:** 10.1002/hsr2.71330

**Published:** 2025-10-06

**Authors:** Hareesha Rishab Bharadwaj, Matan Bone, Dushyant Singh Dahiya, Aditya Gaur, Khabab Abbasher Hussien Mohamed Ahmed, Umar Akram

**Affiliations:** ^1^ Faculty of Biology Medicine and Health The University of Manchester Manchester UK; ^2^ Matan Bone, Salford Royal Hospital Northern Care Alliance NHS Foundation Trust Salford UK; ^3^ Division of Gastroenterology, Hepatology and Motility The University of Kansas School of Medicine Kansas USA; ^4^ Yeovil District Hospital, Somerset NHS Foundation Trust Higher Kingston Yeovil UK; ^5^ Faculty of Medicine University of Khartoum Khartoum Sudan; ^6^ Department of Medicine Allama Iqbal Medical College Pakistan

**Keywords:** cancer epidemiology, healthcare disparities, late‐stage diagnosis, mortality‐to‐incidence ratio, Pancreatic cancer, Sub‐Saharan Africa, treatment access

## Abstract

**Background and Aims:**

Pancreatic cancer remains a significant public health challenge in Sub‐Saharan Africa (SSA), with a high mortality‐to‐incidence ratio driven by late‐stage diagnoses, limited therapeutic access, and systemic healthcare disparities. This perspective aims to synthesize current evidence on the epidemiology, diagnostic capacity, treatment options, and structural barriers contributing to poor outcomes, while highlighting opportunities for targeted interventions and policy reforms.

**Methods:**

We conducted a narrative review of peer‐reviewed literature, regional reports, and epidemiological studies describing the burden and management of pancreatic cancer across SSA. Data were synthesized to characterize diagnostic pathways, surgical and palliative care practices, workforce capacity, and environmental risk factors. Strategies to address identified gaps were developed based on published evidence from similar resource‐constrained settings.

**Results:**

Available data indicate that over 80% of patients in SSA present with advanced or metastatic disease. Diagnostic imaging, including CT and MRI, remains inaccessible in many regions, and biopsies are frequently obtained intraoperatively due to limited endoscopic capacity. Curative surgical resections are rare (< 10%), with palliative bypass procedures predominating. Chemotherapy and radiotherapy services are inconsistently available, and access to opioid analgesics remains below 50% in many centres. Contributing factors include underdeveloped infrastructure, insufficient specialized training, high out‐of‐pocket costs, and heavy metal environmental exposures.

**Conclusion:**

Improving pancreatic cancer outcomes in SSA requires a comprehensive, multi‐sectoral response that prioritizes infrastructure investment, workforce training, and equitable access to diagnostics and therapies. Establishing regional cancer centres, expanding perioperative and palliative nursing roles, strengthening cancer registries, and enforcing environmental regulations are critical steps. Collaboration with international organizations and community stakeholders will be essential to develop context‐specific guidelines and sustainable solutions that can reduce disease burden and improve survival rates in the region.

## 1 Introduction

Pancreatic cancer remains one of the deadliest malignancies worldwide, with its incidence steadily rising. Despite significant advances in molecular biology that have improved our understanding of the disease's pathogenesis, the primary causal factors for pancreatic cancer are still poorly understood [1]. In Sub‐Saharan Africa, the burden of this disease is compounded by multiple challenges, including late‐stage diagnoses, limited access to advanced diagnostic and therapeutic interventions, broader systemic healthcare constraints, and environmental factors such as exposure to heavy metal pollutants and industrial toxins. Additionally, genetic factors influencing treatment response may contribute to disparities in outcomes, particularly within African populations. However, there remains a significant gap in research regarding the prevalence, epidemiology, management, and complications following pancreatic cancer diagnoses in the region [2]. Consequently, this article aims to highlight the limited existing evidence on pancreatic cancer in Sub‐Saharan Africa while calling for more extensive studies to address these gaps.

### 1.1 Current Landscape of Pancreatic Cancer Incidence and Management

The burden of pancreatic cancer in Sub‐Saharan Africa varies significantly between countries. According to recent data, the incidence rate was 0.35 per 100,000 (95% UI, 0.26–0.47), with a death rate of 0.31 (95% UI, 0.23–0.42) and a DALY rate of 14.79 (95% UI, 10.82–19.72). Central Sub‐Saharan African countries exhibited the lowest incidence, death, and DALY rates within the region. Nations such as Ethiopia, Guinea, Niger, Somalia, and Chad recorded some of the lowest figures; however, this is likely due to underreporting and underdiagnosis of pancreatic cancer [3, 4]. A notable characteristic of pancreatic cancer in the region is its high mortality‐to‐incidence ratio (MIR). With one of the highest MIRs at 0.97, the number of deaths nearly equals the number of new diagnoses, underscoring the aggressive nature of pancreatic cancer [3, 4, 5]. This elevated MIR is linked to late diagnoses, limited treatment options, and insufficient healthcare infrastructure, all of which contribute to poor outcomes [6].

The current challenges in pancreatic cancer care across Sub‐Saharan Africa are deeply rooted in longstanding systemic issues. Historically, national health policies have prioritised infectious diseases such as malaria, HIV/AIDS, and tuberculosis, often at the expense of non‐communicable diseases like cancer. This has resulted in chronic underfunding of oncology services and a significant shortage of specialised healthcare professionals [2]. Economically, fragmented procurement systems and low treatment volumes have driven up the costs of cancer medications and equipment, while also complicating quality assurance and supply chain reliability. Infrastructure limitations further exacerbate these problems, as many primary and secondary healthcare facilities lack essential diagnostic tools such as CT scanners, radiotherapy units, or surgical capabilities. Compounding these factors is a widespread absence of robust cancer registries and epidemiological data, which impairs timely diagnosis, resource allocation, and the development of targeted national cancer control strategies [2, 3, 5]. Together, these interrelated constraints have sustained a cycle of late detection, poor treatment outcomes, and minimal policy attention to pancreatic and other gastrointestinal malignancies in the region.

Diagnosing pancreatic cancer at an early, treatable stage remains a significant challenge across Sub‐Saharan Africa. A study in southeast Nigeria assessed 178 pancreatic cancer patients between 2013 and 2021, revealing that 87.1% of patients presented after 6 months [6, 7, 8]. Financial constraints and late referrals were identified as key factors contributing to delayed diagnoses. In Kenya, a study at Kenyatta National Hospital found that pancreatic cancer often presents in younger populations, with a median age of diagnosis of 58.5 years. Over 54% of patients were diagnosed with metastatic disease at the time of presentation [9]. Similar trends have been observed in other Nigerian studies, such as those at the University College Hospital in Ibadan, where pancreatic cancer is increasingly diagnosed in younger adults at advanced stages, with limited curative options available [7]. Furthermore, there is a wide disparity in the availability of diagnostic imaging across the region. While studies in Nigeria indicate sufficient access to abdominal CT imaging, research from Malawi highlights a severe lack of access to timely imaging [10].

Surgical outcomes for pancreatic cancer in Sub‐Saharan Africa exhibit considerable variability across countries, with overall results generally being less favourable compared to high‐income regions. A retrospective cohort study from Nigeria found that 66.3% of pancreatic cancer patients underwent surgery; however, only a small fraction (1.7%) underwent pancreatic resections. The study also revealed a high mortality rate, with 79.8% of patients dying within 18 months of diagnosis. The delayed presentation was significantly correlated with increased mortality [6, 8]. In Ethiopia, a retrospective review of 52 patients diagnosed between 2016 and 2021 showed that 77% of patients received palliative surgeries rather than curative interventions, with notably high morbidity and mortality rates [11]. Additional data from Kenya showed that only 7% of patients underwent radical pancreatoduodenectomy (Whipple procedures), while 57% received palliative operations and 19% had exploratory laparotomies [12]. From Tanzania's Kilimanjaro Christian Medical Centre (2019–2022), 53 patients underwent open bypass (double or triple) for unresectable disease, with a median survival of approximately 65 days; 88% resumed normal oral intake but 22.6% experienced palliation failure [13]. These figures underscore the predominance of palliative rather than curative surgical care in the region. A summary of the above discussion is present in Table 1.

**Table 1:** Estimated prevalence of procedures undertaken for pancreatic cancer in Sub‐Saharan Africa based on available data.

Procedure type	Estimated %	Notes
Pancreaticoduodenectomy (Whipple)	~7%	Only open; no laparoscopic or robotic approaches reported
Palliative bypass (gastrojejunostomy, biliodigestive)	> 50%	Open bypass common (double/triple)
Exploratory laparotomy/biopsy	~19%	Biopsies often obtained intraoperatively
Enteral stenting	< 1%	Rare use (1 patient out of 242)

Minimally invasive approaches, such as laparoscopic or robotic resections, are not yet documented in these settings.

Furthermore, there is a significant paucity of research exploring the efficacy, landscape, and outcomes of chemotherapy and radiotherapy for pancreatic cancer in the region. Studies assessing the quality of life of patients, as well as the experiences of trainees and healthcare professionals, are also virtually non‐existent. This lack of data highlights the urgent need for comprehensive research to evaluate treatment modalities, their outcomes, and the broader impact of pancreatic cancer care in Sub‐Saharan Africa.

Pancreatic care in Sub‐Saharan Africa is fraught with numerous challenges, many of which are exacerbated by systemic healthcare shortcomings across the region. A critical issue is the acute shortage of specialized healthcare professionals, including gastroenterologists, oncologists, and surgeons trained in complex pancreatic procedures. For instance, at present, there exists only 110 gastrointestinal specialists in Nigeria, catering to a population of over 200 million. This workforce gap severely limits early diagnosis and timely access to effective diagnosis and treatment for pancreatic diseases [14]. Diagnostic tools such as imaging modalities and biopsies are frequently unavailable or prohibitively expensive, resulting in delayed diagnoses and treatment initiation. At present, in Eastern Africa, there exists only 0.12 endoscopists per 100,000 population, severely impairing the ability for prompt diagnosis.13 The absence of comprehensive cancer registries and reliable data further hinders the allocation of resources and an accurate understanding of pancreatic cancer prevalence [15, 16, 17, 18].

One of the primary reasons for suboptimal outcomes in complex pancreatic surgeries is the lack of adequately trained surgeons and surgical infrastructure. Many hospitals are ill‐equipped to perform even basic surgical procedures, let alone meet the demands of advanced surgical and anaesthetic requirements [19]. Postoperative mortality rates remain alarmingly high, largely due to the lack of intensive care unit (ICU) facilities for essential postsurgical monitoring and interventions [20, 21]. The availability of essential treatments, such as chemotherapy and radiotherapy, is also severely limited. Chemotherapy drugs like Gemcitabine, which are standard in the treatment of pancreatic cancer globally, are often inaccessible in Sub‐Saharan Africa due to high costs and inconsistent supply chains [22]. Delays in chemotherapy administration lead to the treatment of larger, more advanced tumours and poorer patient outcomes [23]. Radiotherapy services face similar challenges, with insufficient infrastructure, inequitable distribution, long travel distances, and extended waiting times reducing their efficacy. For instance, in countries such as Chad, even basic diagnostic tools for cancer care are unavailable, further delaying or preventing timely intervention [24, 25].

Late‐stage diagnosis of pancreatic cancer often necessitates palliative care, yet symptom management remains inadequate in many regions. Endoscopic palliation, when available, is a valuable adjunct. In a Kenyan cohort, 29.3% of patients underwent endoscopic biliary stenting, 17.3% had open biliodigestive bypass, and 11.6% required percutaneous transhepatic drainage. ERCP with biliary stent placement effectively relieves jaundice in over 80–90% of cases. SEMS outperform plastic stents, offering longer patency (approximately 6–10 months compared to 3–5 months) and fewer re‐interventions. However, EUS‐guided biliary drainage is emerging but remains largely limited to expert centres. Access to essential medications for pain control is also limited, leaving patients to endure significant suffering [26, 27]. The availability of opioid analgesics was less than 50% in many centres across Sub‐Saharan Africa [28]. The overwhelming burden of infectious diseases such as malaria and HIV/AIDS diverts healthcare resources and attention away from non‐communicable diseases like pancreatic cancer, compounding these challenges. Economic constraints pose additional barriers to care. High treatment costs, combined with limited insurance coverage and widespread poverty, render essential diagnostic and therapeutic services inaccessible for a significant proportion of the population [16]. Cost of cancer care is usually five times the annual income in Africa, and most of this is paid out of pocket [29, 30]. Misconceptions and stigma surrounding cancer contribute to delayed healthcare‐seeking behaviours, while awareness and education about pancreatic diseases remain minimal. Geographical disparities in care access further exacerbate inequalities. In countries like Nigeria, where facilities for advanced procedures such as endoscopic retrograde cholangiopancreatography (ERCP) exist, services are largely concentrated in urban centres. This leaves rural populations with little to no access to specialized care.

Environmental factors also increase the risk of pancreatic cancer in the region. Industrial and mining activities release heavy metals such as cadmium, chromium, nickel, and arsenic into the environment, significantly heightening cancer risk in exposed populations [24, 25]. These environmental pollutants, combined with the existing healthcare limitations, underscore the multifaceted challenges in addressing pancreatic cancer in sub‐Saharan Africa.

### 1.2 Future Prospects for Enhanced Care Provision

Future prospects for improving pancreatic cancer care in Sub‐Saharan Africa hinge on addressing the complex, interrelated challenges that currently constrain effective diagnosis, treatment, and palliation. A comprehensive, multi‐sectoral approach will be critical to achieving meaningful progress. One key priority is strengthening healthcare infrastructure. Significant investment is needed to expand the availability of diagnostic imaging, including CT and MRI, as well as pathology services and surgical and oncology facilities. Developing regional cancer centres equipped with multidisciplinary teams could help ensure more equitable access to timely diagnosis and care. Expanding the training of specialized professionals is also essential. Scaling up training programs for surgical oncologists, gastroenterologists, radiologists, and palliative care physicians will build long‐term capacity. Partnerships with academic institutions and professional societies can support knowledge exchange, mentorship, and retention of skilled staff [3, 25].

Robust cancer surveillance systems are urgently needed to track incidence, outcomes, and resource gaps. Enhancing cancer registries and epidemiological research will generate the data necessary to inform policymaking, guide resource allocation, and develop region‐specific treatment guidelines. Given limited surgical and oncology resources, perioperative and palliative nursing training can have a transformative impact by providing continuity of care across the disease trajectory. Specially trained nurse navigators can support patients in the preoperative phase through nutrition optimization, patient education, and proactive pain management planning. In the postoperative setting, nurses can manage central venous catheters and negative pressure wound therapy, detect complications early, and coordinate safe discharge. At the end of life, nursing staff play a vital role in pain management, emotional support, and palliative care. They can also oversee systemic therapy, including chemotherapy administration, opioid prescribing, and enteral feeding tube care. To achieve this, hybrid online and in‐country workshops led by experienced oncology nurse navigators from established cancer centres could be established. Training should focus on perioperative protocols, enteral nutrition, catheter and wound care, pain control strategies, and holistic palliative management [26, 27, 28]. In the long term, these programs can empower local nurses to become trainers themselves, helping sustain improvements in care quality. Leveraging digital health and telemedicine will also be important. Tele‐oncology platforms and virtual multidisciplinary team meetings can link regional hospitals to international expertise, enabling case discussions, remote mentorship, and shared decision‐making. Mobile health solutions can help patients in rural communities track symptoms and access reliable health information.

Improving public awareness and community engagement is another priority. Educational campaigns targeting patients, families, and traditional healers can help counteract stigma, dispel misconceptions about cancer, and encourage earlier presentation to healthcare facilities. Strengthening supply chains and access to essential medicines will require collaboration with governments, NGOs, and pharmaceutical companies to ensure a reliable supply of chemotherapy agents, stents, analgesics (including opioids), and nutritional supplements. Finally, stronger enforcement of environmental regulations is needed to reduce exposure to heavy metals and other carcinogens released by industrial and mining activities, which contribute to the burden of pancreatic and other gastrointestinal malignancies.

Continued research and innovation remains paramount. Emerging nanotechnology strategies—for example, iodine‐containing radiosensitizers—hold promise to improve radiotherapy efficacy in resource‐constrained settings that possess basic radiation facilities. Empirical studies highlight the use of high‐Z element nanomaterials and metal‐organic frameworks to enhance tumour‐targeted radiotherapy and chemotherapy. Khan et al. (2024) further report ferritin‐ and folate‐functionalized iron‐based MOFs as drug carriers with strong cytotoxicity in vitro [29, 30, 31, 32, 33]. Although these remain experimental, they point toward future therapeutic innovations even in low‐resource environments. With concerted action across these domains, it is possible to progressively reduce the burden of pancreatic cancer in Sub‐Saharan Africa. By expanding the workforce, strengthening health systems, and empowering nurses and allied professionals, future efforts can improve early detection, optimize perioperative and palliative care, and ultimately save lives. The above is summarised in Table 2.

**Table 2:** Existing challenges and proposed strategic interventions.

Challenge	Evidence/Context	Recommended strategy
Late‐stage referrals & cost barriers	> 80% present at advanced stage; high out‐of‐pocket costs	Implement insurance schemes, community outreach, early detection campaigns
Limited imaging & biopsy capacity	< 50% CT/MRI availability; biopsy via laparotomy common	Invest in mobile imaging; train staff in percutaneous and endoscopic biopsy
Low curative surgery rates	< 10% undergo Whipple resection; palliative bypasses dominate	Develop regional HPB centres, train surgeons, support perioperative nurse navigation
Inadequate chemo/radiotherapy	Gemcitabine/radiotherapy largely unavailable	Improve drug distribution logistics; expand radiotherapy facilities
Poor palliative care/opioid scarcity	Opioids accessible in < 50% of centres	Expand training, regulatory reform, importation of essential analgesics
Environmental carcinogen exposure	Heavy metal pollution in mining zones	Enforce regulations, environmental monitoring, corporate responsibility programs
Data gaps and weak registries	Cancer registries incomplete or non‐existent	Strengthen national registries, support epidemiological research, data‐sharing networks

## 2 Conclusion

In summary, pancreatic cancer in Sub‐Saharan Africa remains a profoundly challenging disease characterized by delayed diagnosis, limited treatment options, and high mortality. Addressing these disparities requires coordinated investments in infrastructure, workforce training, cancer registries, and community education, alongside innovative strategies adapted to regional constraints. With sustained commitment and collaborative efforts, it is possible to improve outcomes and reduce the burden of this devastating malignancy across the region.

## Author Contributions

**Hareesha Rishab Bharadwaj:** conceptualization, methodology, writing – original draft, writing – review and editing. **Matan Bone:** writing – original draft, writing – review and editing. **Dushyant Singh Dahiya:** writing – original draft, writing – review and editing, supervision. **Aditya Gaur:** writing – original draft, writing – review and editing. **Khabab Abbasher Hussien Mohamed Ahmed:** writing – original draft, writing – review and editing, supervision. **Umar Akram:** Writing – original draft, writing – review and editing, supervision.

## Ethics Statement

The authors have nothing to report.

## Conflicts of Interest

The authors declare no conflicts of interest.

## Suggested Reviewers

1. Prof. Athanasios Alexio,

Department of Science and Engineering, Novel Global Community Educational Foundation, Hebersham, NSW 2770, Australia; alexiou@ngcef.net

2. Prof. Vladyslav Sikora,

Department of Clinical and Experimental Medicine, University of Foggia, Via Napoli, 20, Foggia, 71122, Italy, V.sikora@med.sumdu.edu.ua

## Transparency Statement

The lead author Khabab Abbasher Hussien Mohamed Ahmed affirms that this manuscript is an honest, accurate, and transparent account of the study being reported; that no important aspects of the study have been omitted; and that any discrepancies from the study as planned (and, if relevant, registered) have been explained.

## Data Availability

Data sharing not applicable to this article as no datasets were generated or analysed during the current study.
